# Hepatocardiac or Cardiohepatic Interaction: From Traditional Chinese Medicine to Western Medicine

**DOI:** 10.1155/2021/6655335

**Published:** 2021-03-12

**Authors:** Yaxing Zhang, Xian-Ming Fang

**Affiliations:** ^1^Department of Physiology, School of Basic Medical Sciences, Guangzhou University of Chinese Medicine, Guangzhou, Guangdong, China; ^2^Issue 12 of the Master-Apprentice Education of Guangxi Traditional Chinese Medicine, College of Continuing Education, Guangxi University of Chinese Medicine, Nanning, Guangxi, China; ^3^Department of Cardiology, Ruikang Hospital Affiliated to Guangxi University of Chinese Medicine/Ruikang Clinical Faculty of Guangxi University of Chinese Medicine/Guangxi Hospital of Integrated Chinese and Western Medicine, Guangxi University of Chinese Medicine, Nanning, Guangxi, China

## Abstract

There is a close relationship between the liver and heart based on “zang-xiang theory,” “five-element theory,” and “five-zang/five-viscus/five-organ correlation theory” in the theoretical system of Traditional Chinese Medicine (TCM). Moreover, with the development of molecular biology, genetics, immunology, and others, the Modern Medicine indicates the existence of the essential interorgan communication between the liver and heart (the heart and liver). Anatomically and physiologically, the liver and heart are connected with each other primarily via “blood circulation.” Pathologically, liver diseases can affect the heart; for example, patients with end-stage liver disease (liver failure/cirrhosis) may develop into “cirrhotic cardiomyopathy,” and nonalcoholic fatty liver disease (NAFLD) may promote the development of cardiovascular diseases via multiple molecular mechanisms. In contrast, heart diseases can affect the liver, heart failure may lead to cardiogenic hypoxic hepatitis and cardiac cirrhosis, and atrial fibrillation (AF) markedly alters the hepatic gene expression profile and induces AF-related hypercoagulation. The heart can also influence liver metabolism via certain nonsecretory cardiac gene-mediated multiple signals. Moreover, organokines are essential mediators of organ crosstalk, e.g., cardiomyokines link the heart to the liver, while hepatokines link the liver to the heart. Therefore, both TCM and Western Medicine, and both the basic research studies and the clinical practices, all indicate that there exist essential “heart-liver axes” and “liver-heart axes.” To investigate the organ interactions between the liver and heart (the heart and liver) will help us broaden and deepen our understanding of the pathogenesis of both liver and heart diseases, thus improving the strategies of prevention and treatment in the future.

## 1. Introduction

In the theoretical system of Traditional Chinese Medicine (TCM), there are close relationships/interactions between the liver and heart (the heart and liver) according to the first records in the original literature Huang-Di-Nei-Jing (The Yellow Emperor's Canon of Medicine), the earliest existing TCM classics, which summarized the medical achievements and treatment experience before China Spring and Autumn, Warring State Period (770 B.C.–221 B.C.). Huang-Di-Nei-Jing established the unique theoretical system of TCM and became the basis of TCM. Moreover, the “five-zang (also known as five-viscus or five-organ) correlation theory, 五脏相关学说/理论” established by the National Chinese Medical Science Master (Guo-Yi Master) Deng Tietao (邓铁涛) in the 1960s also contains the “liver-heart correlation theory (or heart-liver correlation theory)” [1–4]. These interactions between the liver and heart (the heart and liver) have been extensively used by doctors of TCM to guide the clinical diagnosis, prevention, and treatment of both hepatic and cardiovascular diseases (CVDs) for more than 2200 years in China.

In Modern Medicine (Western Medicine), most notably from Claude Bernard in the 19th century, it is first suggested that a system involving chemical messengers ensures the communication between the different organs of the body [5]. Since the discovery of cardiac natriuretic peptides (NPs) by de Bold in 1981, it is well known that the heart has an endocrine function [6, 7]. Now, the biological and medical scientists found that the heart can secrete other proteins besides NPs, they are termed as “cardiomyokines” [8, 9], and the liver is also an endocrine organ that secretes “hepatokines” [10–12]. These “organokines” are essential mediators of organ interaction between the liver and heart (the heart and liver) [8, 10, 11, 13–16].

In modern anatomy and physiology, the heart is central to hemodynamics of many organs both in the form of distributing the oxygenated blood and delivering deoxygenated blood in order to send it to the lungs [17]. The liver, which has high metabolic activities, receives up to 25% of cardiac output, coming by two systems of blood vessels: the hepatic artery and the portal vein [18]. The venous drainage occurs by hepatic veins and the inferior vena cava, which have no valves, resulting in direct transmission of the rise of right heart filling pressures to the liver [18]. Moreover, accumulating basic and clinical evidences indicate that acute as well as chronic heart disease can directly contribute to an acute or chronic worsening of liver function and vice versa [19].

Therefore, there exist the essential interorgan crosstalk between the heart and liver (the liver and heart). The aim of this review is to comprehensively summarize and discuss the hepatocardiac or the cardiohepatic interaction from the perspective of TCM and Western/Modern Medicine. These will broaden and deepen our understanding of the hepatic and cardiovascular physiology, and the pathogenesis of liver diseases and heart diseases, thus helping us improve the prevention and treatment strategies in the future.

## 2. Hepatocardiac or Cardiohepatic Interaction in Traditional Chinese Medicine

### 2.1. The Physiological Relationship between the Liver and Heart Based on “Zang-Xiang Theory”

The words “zang-xiang” were firstly recorded in the TCM literature Su wen·Liu jie zang xiang lun, which is a section of Huang-Di-Nei-Jing. The word “zang” refers to the internal organs hidden in the body, while “xiang” refers to the physiological and pathological phenomena that appear outside. The “zang-xiang theory” investigates the physiological functions and pathological changes of “zang-fu” and their relationship. Based on the physiological function of “zang-fu,” they are divided into “five-zang” (liver, heart, spleen, lung, and kidney), “six-fu” (gallbladder, small intestine, stomach, large intestine, bladder, and san-jiao), and “qi-heng-zhi-fu” (brain, marrow, bone, vessels (mai), gallbladder, and nv-zi-bao (also known as the uterus)). Among these, the heart is considered as the “official of monarch” according to the TCM literature Su wen·Ling lan mi dian lun. Heart governs the blood and vessels (mai, also known as “xin zhu xue-mai”) and controls “shen-zhi,” also known as heart controlling “shen-ming” or heart storing “shen.” Generally, “shen” refers to the external performance of the life activities of the whole human body. Narrowly, “shen” refers to spirit, consciousness, and thinking activities. The liver is considered as the “organ of general” according to the TCM literature Su wen·Ling lan mi dian lun, physiologically, the liver governs “shu-xie,” also known as “liver-governing free flow of Qi” or “liver-controlling dispersion,” and the liver is also capable of storing blood and regulating the distribution of blood volume in all parts of the human body and capable of storing soul based on “liver storing blood and blood housing soul.” Therefore, there is a functional relationship between the heart and liver (the liver and heart) mediated by “blood movement” and “qing-zhi regulation.”

### 2.2. The Relationship between the Liver and Heart Based on “Five-Element Theory”

The “five-element theory” in TCM refers to the “zang-fu,” “five-guan,” “five-ti,” “five-ye,” “five-zhi,” and “jing-luo (meridians and collaterals),” and others of the human body into the five elements: wood, fire, soil/earth (known as “Tu” in Chinese), gold (known as “Jin” in Chinese), and water. The application of “five-element theory” in TCM is mainly to analyze and study the five-element attribute of “zang-fu,” “jing-luo,” and others according to the characteristics of five elements, to investigate the relationships in “zang-fu,” in “jing-luo” and in others, and in their physiological functions based on the “generation-inhibition” of five elements, to explain the mutual influence of the diseases according to the “cheng-wu” (“cheng,” domineer over the weak by being strong; “wu,” reverse restriction in five elements) of five elements. Therefore, the “five-element theory” in TCM is not only used as a theoretical exposition but also has the significance in clinical practices.

**Figure 1:**
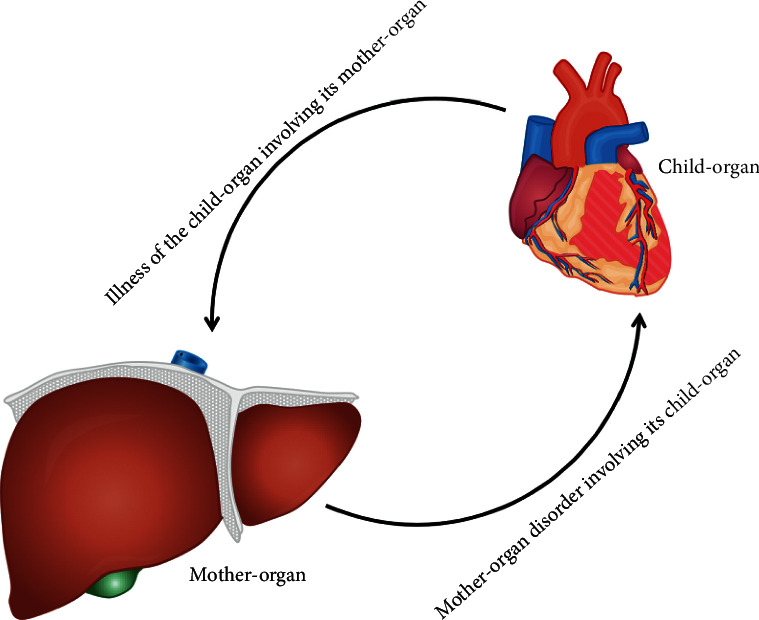
The disturbance of liver-heart homeostasis in TCM. In TCM, the liver is the mother-organ and the heart is the child-organ. The mother-child relationship of the liver and heart is essential for modulating both organs' homeostasis. The “mother-organ disorder involving its child-organ” refers to the transformation of the disease from the mother viscera to the child viscera; therefore, liver diseases may trigger heart diseases. The “illness of the child-organ involving its mother-organ” indicates that the diseases can be transformed from the child viscera to the mother viscera; therefore, heart diseases may induce liver diseases. For example, the insufficiency of heart blood involves the liver and induces blood deficiency of the liver, thus forming “heart-liver blood deficiency.” The effulgent heart fire conversely involves the liver and triggers liver fire, thus inducing “hyperactivity of heart fire and liver fire.”

In this theory, the liver belongs to wood as the mother of heart, and the heart belongs to fire as the son of liver; therefore, the “wood inducing fire” is equaled to the “liver inducing heart.” The “mother-child relationship of the liver and heart” is a physiological description of mutual promotion and mutual restriction relationship in the liver and heart according to “five-element theory” in TCM [20]. If the “mother-child relationship of the liver and heart” was destroyed, the pathological phenomena of “mother-organ disorder involving its child-organ” (which indicates that liver disease may induce heart disease) and “illness of the child-organ involving its mother-organ” (which indicates that heart disease may induce liver disease) would occur [20] (Figure 1). Thus, the liver and heart influence each other under normal or pathophysiological conditions.

### 2.3. Liver and Heart in “Five-Zang Correlation Theory”

The five visceral systems are interrelated, which is one of the basic characteristics of TCM academic ideologies since the ancient times in China, for example, “five-zang xiang-tong” recorded in Su wen·Yu ji zhen zang lun, “five-zang diseases theory” by Zhang Zhongjing (China Han Dynasty), “five-yun zhu-bing/diseases” by Liu Wansu (China Jin Dynasty), “five-element hu-han” recorded in Fu-xing-jue (Dunhuang Legacy), “five-zang pang-tong” by Sun Simiao (China Tang Dynasty), “five-zang chuan-zao” by Li Ting (China Ming Dynasty), “five-element hu-cang” by Zhang Jiebin (China Ming Dynasty), “five-zang mutually guan-she” by He Mengyao (China Qing Dynasty), and others [21]. Based on the classic “five-element theory,” the theories mentioned above, and the long-term clinical practice, Deng Tietao established the “five-zang correlation theory” in 1961 [3]. The theory refers to that in the large system of the human body, the “five-zang” and their corresponding “six-fu,” limbs, skin, hair, tendons (jin), vessels (mai), meat, five-guan, nine orifices, etc. constitute the five visceral systems, and there are horizontal, vertical, and cross multidimensional connections within the visceral system, between the visceral system and the visceral system, between the visceral system and the human body system, between the visceral system and the nature and society [1, 2]. They promote and restrict each other in order to play different functions and coordinate the normal activities of the body [1]. Moreover, the five visceral systems interact with each other under the pathological conditions [1]. In short, the “five-zang” organs are related [1], which highlight the importance of “five-zang” system communication in modulating body homeostasis; for example, the coronary heart disease is related to “Qi deficiency of five-zang” [22] and can be treated by “Yi-Qi-Chu-Tan-Fang” [23].

Based on zang-xiang, yin-yang, five-element, qi-blood, jing-luo, and qing-zhi (seven emotions) theory according to Huang-Di-Nei-Jing and based on “five-zang correlation theory” by Deng Tietao, there are close physiological and pathological relationships/interactions between the liver and heart (the heart and liver) in TCM theories [1–4]. Clinically, these TCM theories have been extensively used to treat both the heart diseases and liver diseases. For example, the methods of “Shu-Liver, 疏肝” and “Rou-Liver, 柔肝” based on “treating the heart disease from the liver” have been extensively used for treating coronary heart disease [24–26], and the methods of “Xing-Qi-Huo-Xue, 行气活血” and “Bu-Xue, 补血” have been used for treating nonalcoholic fatty liver disease (NAFLD) [27–30]. These connections between the liver and heart (the heart and liver) have also been confirmed by the Western Medicine.

## 3. Organ Interaction in Western Medicine

**Figure 2:**
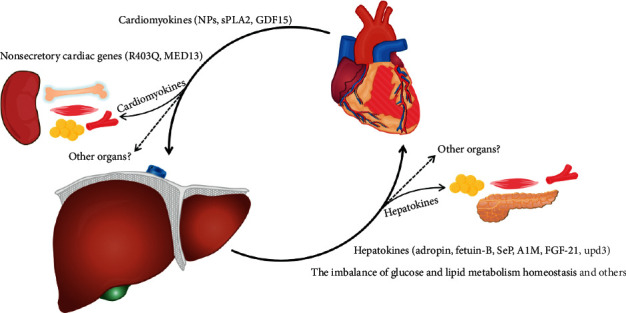
The modern molecular biological basis of liver-heart axis and heart-liver axis. The liver is sensitive to hemodynamic changes, the liver and heart are connected via blood circulation, pathologically, certain liver disease may cause heart diseases and vice versa. In molecular biology, cardiomyokines secreted from cardiomyocytes, such as natriuretic peptides (NPs), secreted phospholipase A2 (sPLA2), and growth differentiation factor 15 (GDF15), link the heart to liver. Besides the heart, blood vessels, and liver, several cardiomyokines also target other remote organs, for example, kidneys, bone, skeletal muscles, and adipose tissues. The nonsecretory cardiac genes, such as familial hypertrophic cardiomyopathy-(HCM-) causing mutation in myosin (R403Q) and Mediator complex subunit 13 (MED13), may also influence liver metabolism. Conversely, the hepatokines, for example, adropin, fetuin-B, selenoprotein P (SeP), *α*1-microglobulin (A1M), fibroblast growth factor-21 (FGF-21), and unpaired 3 (upd3), act as novel linkers connecting the liver to heart. Additionally, some hepatokines also link the liver to adipose tissue, skeletal muscle, blood vessels, pancreas, and others. Moreover, the imbalance of hepatic glycolipid metabolism homeostasis, as well as other factors involved in liver diseases, may also contribute to cardiovascular diseases (CVDs).

Organ interaction, also known as organ crosstalk or interorgan communication, can be defined as the complex and mutual biological communication between different tissues/organs of multicellular organisms via multiple signals [5, 31]. Normally, the maintenance of systemic homeostasis and the adaption to external conditions, such as nutritional and environmental challenges, require a finely tuned system of interorgan communication; however, sudden or chronic dysfunction in any organ causes dysregulation in another organ [5, 31–33]. Interorgan communication has been shown to play essential roles in orchestrating metabolic health [5, 33]. Mechanistically, bioactive peptides and proteins (e.g., hormones and cytokines), extracellular vesicles (EVs, e.g., exosome and migrasome), and certain nonsecretory genes are the key messengers in modulating the interorgan communication [5, 34–43]. Moreover, organokines are the novel players mediating the interorgan communication, they are proteins exclusively or predominantly produced by and secreted from a specific tissue (e.g., the functional proteins released from adipose tissue are termed as “adipokines” and skeletal muscle-derived proteins are known as “myokines”), but they are not simply markers of the function of their source tissue, and all organokines have the paracrine or endocrine actions, or both [13]. Similar to that in TCM, the interorgan communication of Western Medicine also supports the organ interaction between the liver and heart (the heart and liver), and there exist the liver-heart axis and the heart-liver axis (Figure 2).

### 3.1. The Liver-Heart Axis in Modern Medicine

#### 3.1.1. Liver Diseases Affecting the Heart

The close interaction and connection between the cardiac and hepatic functions are well known, for example, “hepatic/cirrhotic cardiomyopathy” is an important clinical entity which best describes the mutual pathogenical influence between these two organs [44]. Patients with end-stage liver disease (liver failure/cirrhosis) displayed hyperdynamic circulation characterized by low systemic vascular resistance and high cardiac output state [45, 46]. However, the cardiac response to physiological, pathophysiological, or pharmacological stimuli (such as exercise, hemorrhage, infection, and surgery) is abnormal with systolic and diastolic dysfunction, as well as electromechanical abnormalities in the absence of other known causes of cardiac disease, a condition termed “cirrhotic cardiomyopathy” [45, 47–53]. Moreover, in patients with liver cirrhosis, the elevated parameters of myocardial edema and fibrosis were observed at magnetic resonance imaging (MRI), these were more abnormal with greater severity of liver disease [54].

Bile duct ligation (BDL)-induced advanced liver fibrosis is a suitable mouse model to investigate the pathophysiology of hepatic/cirrhotic cardiomyopathy at a preclinical level, as it resembles the characteristics of the clinical syndrome seen in patients [55]. One of the main contributors to the BDL-induced liver fibrosis is tissue inflammation, which contributes, as liver failure develops, to the production and excretion of several inflammatory cytokines, such as tumor necrosis factor-alpha (TNF-*α*), into the blood, culminating in a general inflammatory response and subsequent oxidative stress, and the heart is one of the major organs involved [55, 56]. Cannabinoid-2 receptor (CB_2_-R), a negative regulator of ischemia/reperfusion (I/R)-induced liver injury and carbon tetrachloride-induced hepatic fibrosis, is upregulated in the liver and heart of BDL mice [55, 57–59]. Treatment BDL mice with a selective CB_2_-R agonist HU910 alleviated hepatic inflammation and fibrosis, restored the hepatic microcirculation, reduced serum levels of TNF-*α*, and improved cardiac dysfunction, myocardial inflammation, and oxidative stress [55]. These beneficial effects of HU910 indicated that controlling the liver and/or myocardial inflammation may delay or prevent the development of cardiomyopathy in severe liver disease [55]. Thus, the liver-heart inflammatory axis has a pivotal pathophysiological role in the pathogenesis of hepatic cardiomyopathy [55].

The liver is a central hub for lipid metabolism and endogenous glucose production; therefore, the liver is crucial for systemic glucose and lipid homeostasis [60]. Fatty liver disease (FLD), which primarily includes alcoholic liver disease (ALD) and NAFLD, encompasses a broad spectrum of pathological changes of the liver, ranging from simple steatosis to steatohepatitis, and liver fibrosis, with eventual progression to cirrhosis and hepatocellular carcinoma (HCC) [61–64]. Moreover, FLD is also associated with extrahepatic manifestations, such as fatal and nonfatal CVDs, leading to an increased morbidity and mortality [65–69]. Although debate continues over the causal relationship between NAFLD and CVDs, many mechanistic and longitudinal studies have indicated that NAFLD is one of the major driving forces for CVDs, and NAFLD should be recognized as an independent risk factor for CVDs apart from other metabolic disorders [66–68, 70–72]. Mechanistically, dysfunction of the glucose and lipid metabolism, activation of low-grade systemic inflammation and oxidative stress, disturbance of immunologic and neuroendocrine homeostasis, activation of the prothrombotic system, intestinal dysbiosis, and some genetic and epigenetic factors for liver diseases, such as NAFLD, may contribute to CVDs [65, 66]. Therefore, the contribution of FLD to CVDs also establishes the liver-heart axis.

#### 3.1.2. Hepatokines Link the Liver to Heart

Besides the mentioned mechanisms above, changes in protein secretions from the fatty liver also contribute to the pathogenesis of CVDs [16, 65, 67, 73–76]. The liver has recently been recognized as an endocrine organ that secretes hepatokines, which are proteins secreted by hepatocytes that can influence metabolic processes through autocrine, paracrine, and endocrine signaling [10–12]. The hepatocyte protein secretome undergoes marked changes in response to liver steatosis, for example, Meex et al. have identified 168 hepatokines, of which 32 were differentially secreted in steatotic versus nonsteatotic hepatocytes, thus promoting insulin resistance and other metabolic complications [11, 77]. Increased intrahepatic levels of triglyceride (TG) induce the changes in hepatokine transcription and endoplasmic reticulum processing, leading to decrease the secretion of some hepatokines (such as sex hormone-binding globulin (SHBG), angiopoietin-like protein 4 (ANGPTL4), and adropin) during steatosis and increase the secretion of other hepatokines (such as fetuin-A, fetuin-B, hepassocin, leukocyte cell-derived chemotaxin 2 (LECT2), retinol-binding protein 4 (RBP4), and selenoprotein P (SeP)) [11]. Key hepatokines can induce either negative (fetuin-A, fetuin-B, hepassocin, LECT2, RBP4, and SeP) or positive (SHBG, fibroblast growth factor-21 (FGF-21), ANGPTL4, and adropin) metabolic effects [11, 78]. In addition to signaling to hepatocytes, most importantly, hepatokines function systemically through transporting to and communicating with distant target tissues, including the skeletal muscle, adipose tissue, pancreas, blood vessels, and heart [10, 11, 13, 16, 79] (Figure 2, right).

Adropin is a nutritionally regulated peptide hormone, secreted primarily by the liver and brain, and it is central to the control of cardiac fuel metabolism [80, 81]. Its expression was declined in the liver with genetically induced obesity or high-fat diet (HFD)-induced obesity, transgenic overexpression or systemic adropin treatment protects against hepatic steatosis, and hyperinsulinemia associated with obesity; therefore, adropin acts as a positive factor governing glucose and lipid homeostasis [80, 81]. Altamimi et al. showed that adropin has an important role in regulating cardiac energy substrate preference through enhancing insulin signaling, stimulating glucose oxidation and inhibiting fatty acid oxidation in the heart of C57Bl/6 mice [82]. They proposed signaling pathways that are modulated by adropin: adropin, possibly via a plasma membrane receptor, such as G-protein coupled receptor 19 (GPR19) or some other mediators, reduces pyruvate dehydrogenase kinase 4 (PDK4) protein levels and stimulates ERK1/2 MAPK, which is also known to regulate PDK4 expression, resulting in a decrease in the inhibitory phosphorylation of pyruvate dehydrogenase (PDH), the rate limiting glucose oxidation enzyme, leading to its activation and enhancement of glucose oxidation [82]. On the other hand, adropin treatment appears to reduce JNK phosphorylation, which otherwise inhibits insulin receptor substrate 1 (IRS-1) signaling, thus resulting in an overall stimulation of insulin signaling including phosphorylation of Akt (protein kinase B), FOXO1 (forkhead box O1, further reduces PDK4 expression), and AS160 (Akt substrate of 160 kDa, increases glucose transporter 4 (GLUT4) plasmalemmal translocation and glucose uptake), and inhibitory phosphorylation of GSK3*β* (glycogen synthase kinase 3 beta, enhances glycogen synthesis) [82]. All these above lead to a net enhancement of insulin sensitivity and glucose metabolism and utilization [82]. The beneficial effect of adropin exposure on the impaired cardiac glucose oxidation was also confirmed in prediabetic obese mice under HFD conditions [83]. In detail, adropin reduces the expression of mitochondrial acetyltransferase enzyme general control of amino acid synthesis 5 like 1 (GCN5L1), which results in decreasing the fuel metabolism enzyme PDH lysine acetylation, thus increasing the activity of PDH to favor cardiac glucose utilization in HFD-induced prediabetic obese mice [83]. In addition, adropin has the antiatherosclerotic effects by suppressing monocyte-endothelial cell adhesion and smooth muscle cell proliferation [84]. These studies all used the exogenous adropin to evaluate its role in the heart; however, the physiological and pathological roles of endogenous adropin in heart homeostasis still need further investigation.

*α*1-Microglobulin (A1M) is a 26 kDa plasma and tissue protein, which is mainly synthesized in the liver, but also in smaller amounts in peripheral organs [85–87]. A cross-sectional study showed a significant association between urinary A1M-creatinine ratio and NAFLD [88]. Hakuno et al. have identified A1M as an Akt-activating hepatokine by screening the effects of conditioned media on doxorubicin- or hypoxia-induced cardiomyocytes stress in vitro, and the in vivo study also confirmed that A1M is produced by the liver rather than the heart [89]. A1M is secreted into the blood stream, from the liver, and found in blood as complexes with IgA, albumin, and prothrombin (1 *μ*M) and in its free form (1 *μ*M) [86, 90, 91]. After secretion from the liver, A1M is transiently distributed in the infarct and border zones via infiltrated macrophages (MQs) and cardiac fibroblasts during the acute phase of mouse myocardial infarction (MI) [89]. Functionally, A1M enhances MQs migration as well as the proinflammatory response in cardiac fibroblasts and MQs in vitro, intramyocardial administration of recombinant murine A1M augment MQs infiltration, inflammation, and matrix metalloproteinase-9 (MMP-9) mRNA expression in the infarct and border zones, disturbs fibrotic repair, and drives cardiac rupture during the acute phase of MI in vivo [89]. These actions of A1M were partly mediated by its binding to phosphatidic acid (PA) [89]. Therefore, short-term, systemic delivery of CU-3, a selective inhibitor of diacylglycerol kinase alpha (DGK*α*) mediated PA biosynthesis, reduced MQs infiltration, inflammation, and MMP activity during the acute phase, and further mitigated left ventricular remodeling during the chronic phase in mouse MI [89]. This study indicates that targeting hepatic A1M expression and the MQs A1M signaling could be the promising options to mitigate adverse left ventricular remodeling in MI.

SeP (encoded by SELENOP in humans) contains ten selenocysteine residues and functions as a selenium supply protein, and it is primarily produced and secreted by liver [92–95]. SeP causes insulin resistance, at least partly, by dephosphorylating adenosine monophosphate-activated protein kinase (AMPK) [92]. A recent study using SeP knockout (KO) mice and hepatic overexpression of SeP in SeP KO mice indicated that the endogenous SeP mediates the deleterious effect of myocardial I/R injury [96]. SeP gene deletion reduces I/R-induced myocardial apoptosis by increasing the phosphorylation of insulin-like growth factor 1 (IGF1) receptor, Akt, ERK, and S6K, which were reversed by overexpressing SeP in the liver of SeP KO mice [96]. Thus, SeP serves as a hepatokine that contributes to myocardial I/R injury.

Fetuin-B, also a secreted hepatocyte factor, was upregulated in humans with liver steatosis and patients with type 2 diabetes, and it impairs insulin action in myotubes and hepatocytes and causes glucose intolerance in mice, while silencing of fetuin-B in obese mice improves glucose tolerance [77]. The increased expression of fetuin-B in diabetic liver exacerbates myocardial I/R injury and cardiac dysfunction, while suppression of fetuin-B exerts cardiac protective effects [16]. Mechanistically, fetuin-B interacts with insulin receptor-*β* subunit, impairs cardiac insulin signaling, and consequently causes myocardial I/R injury [16]. Therefore, this study confirmed that fetuin-B is a novel linker from the liver to heart I/R injury.

In contrast to the disturbed effects of hepatic A1M, SeP, and fetuin-B for heart repair after myocardial I/R injury, FGF-21 is beneficial for cardiac repair after MI [97]. In 2000, murine and human FGF-21 were identified by Nishimura et al., and they found that FGF-21 mRNA was most abundantly expressed in the liver and also expressed in the thymus at lower levels [98]. Studies in the past few years indicated that FGF-21 is also synthesized in several other tissues, such as pancreas, skeletal muscle, and adipose tissue [99–103]. The pharmacological effects of FGF-21 are mediated by both its central and peripheral actions and by its fine-tuning of interorgan metabolic crosstalk [78]. Recently, during investigating the cardiac effect and mechanism of interleukin (IL)-22 after MI, Tang et al. found that IL-22 promoted hepatocyte-derived FGF-21 production depending on hepatic signal transducer and activator of transcription-3 (STAT-3) activation [97]. Subsequently, FGF-12 arrived at the heart and bound its functional receptor fibroblast growth factor receptor 1 (FGFR-1) in cardiomyocytes, thus modulating the expression of genes that are involved in cholesterol homeostasis, DNA repair, peroxisome, oxidative phosphorylation, glycolysis, apoptosis, and steroid responses, all of which contributed to the survival of cardiomyocytes [97]. Therefore, hepatic STAT3-FGF-21 axis modulated by IL-22 contributes to liver-heart crosstalk and is an essential mechanism for injury repair after MI [97].

Recently, Huang et al. demonstrated an IL-6-like proinflammatory cytokine unpaired 3 (upd3) expressed in *Drosophila* oenocytes (a hepatocyte-like tissue) mediated interorgan communication between the liver and heart [104]. They found that the impaired peroxisomal import in aged *Drosophila* oenocytes promotes ROS production and JNK activation and then induces upd3 as a peroxikine in aged oenocytes, this peroxikine signals to the heart and nonautonomously activates the JAK-STAT pathway in cardiomyocytes, and thus, it causes arrhythmia [104]. This study indicates that peroxisome is a central regulator of inflammaging and liver-heart communication via mediating hepatic peroxikine (also can be termed as “hepatokine”) production. However, the roles of hepatic peroxisomes in age-related heart diseases in human and other mammalian animals are not clear.

Therefore, hepatokines act as essential linkers from the liver to heart in the pathological conditions not limited to NAFLD-related CVDs, but also in myocardial I/R injury and in ageing-related heart diseases, thus establishing a set of liver-heart axes.

### 3.2. The Heart-Liver Axis in Modern Medicine

#### 3.2.1. Heart Diseases Affecting the Liver

Heart failure leads to a chronic inability to meet metabolic requirements of the end organs or skeletal muscle; therefore, the syndrome of heart failure is characterized by organ crosstalks, for example, the well-established “cardiorenal syndrome” [17, 105]. The liver is an organ sensitive to hemodynamic changes, and hepatic involvement in the form of cardiohepatic interaction has been described in patients with acute and chronic heart failure [17, 18, 52, 53]. The mechanisms underlying the cardiac hepatopathy are reduced arterial perfusion, whose deleterious effects are amplified by concomitant hypoxia, and passive congestion secondary to increased systemic venous pressure [53]. The arterial hypoperfusion predominates in acute heart failure leading to hypoxic hepatitis, while chronic passive congestion prevails in congestive hepatopathy secondary to chronic heart failure, and the chronic passive congestion leads to sinusoidal hypertension, centrilobular fibrosis, and ultimately, cirrhosis (“cardiac cirrhosis”) and HCC after several decades of ongoing injury [53, 106]. These forward and backward factors often coexist and potentiate each other [53].

Atrial fibrillation (AF) is the most common among the severe cardiac arrhythmias, which is associated with a high risk of thromboembolism and stroke [107]. AF activates the coagulation system, leading to prothrombotic or hypercoagulable state [107, 108]. The liver is an essential organ synthesizing many coagulation factors and other prothrombic molecules [108]. Using rapid atrial pacing (RAP) rat model, Yaegashi et al. found that short-term RAP mimicking paroxysmal AF markedly altered the hepatic gene expression profile, and hepatic mRNA levels of prothrombic molecules, including fibrinogen chains, prothrombin, coagulation factor X, and antithrombin III, were augmented by short-term RAP [108]. The activation of the IL-6/STAT3 signaling pathway is responsible for the augmented fibrinogen and coagulation factor X production by RAP [108]. Therefore, IL-6 neutralizing antibody pretreatment inhibited RAP-mediated hepatic STAT3 phosphorylation and fibrinogen and coagulation factor X expression [108]. This indicated that the cardiohepatic interactions are also involved in AF-related hypercoagulation.

#### 3.2.2. Cardiomyokines Link the Heart to Liver

The breakthrough discovery of cardiac NPs by de Bold provided the first direct evidence that the heart has an endocrine function [6, 7]. They found that the extracts derived from atrial muscle (atrial natriuretic peptide, ANP) caused a rapid, more than 30-fold increase of sodium and chloride excretions, while urine volume rose 10-fold, and potassium excretion doubled [7]. They concluded that the atrial extract contained an extremely powerful inhibitor of renal tubular NaCl reabsorption [7]. In 1988, Sudoh et al. discovered a new natriuretic peptide of 26 amino-acid residues in porcine brain, eliciting possesses diuretic-natriuretic (e.g., increase in urine output, Na^+^, K^+^, and Cl^−^ excretion) and hypotensive (decrease in mean blood pressure) responses similar to that of ANP, and they have designated the peptide “brain natriuretic peptide” (BNP) [6, 109], which is also localized in the secretory granules of human atrium that contain ANP [110]. Therefore, cardiac ANP- and BNP-mediated communications between the heart and kidney are essential for maintaining sodium and volume homeostasis in health and disease [6, 7, 111].

An emerging concept is that the heart not only regulates blood pressure homeostasis and water-salt balance but also acts as a regulator of whole body metabolism [14, 112–115]. The cardiac NPs are modulators of metabolism, and they induce human fat cell lipolysis and the “browning” of white adipocytes, favor blood glucose control and insulin sensitivity by increasing glucose uptake in human adipocytes, and enhance mitochondrial oxidative metabolism and fat oxidation in human skeletal muscle [14, 116–119]. Moreover, ANP was shown to increase hepatic gluconeogenesis and inhibit glycolysis, in part by inhibiting pyruvate kinase activity, and the effects of ANP are mediated via activation of guanylyl cyclase-linked ANF receptors which elevate cGMP production [15]. It is clear that ANP can protect against hepatic I/R injuries [120, 121]. On the molecular mechanism, ANP activates cGMP-dependent heat shock protein 70 (HSP70) expression and correlates with enhanced binding of HSP70 to inhibitory factor kappa B (I*κ*B) [122], thus attenuating the activation of the proinflammatory transcription factor nuclear factor kappa B (NF-*κ*B) and the expression of TNF-*α* [123, 124]; ANP also attenuates necrotic (mainly in hepatocytes and endothelial cells) and apoptotic (mainly in hepatocytes) cell death [125–127]; ANP increases the phosphorylation of p38 MAPK during liver I/R [128–130]. However, a p38 MAPK inhibitor fails to abolish ANP-mediated antiapoptotic action in the cold I/R liver [126]; the antiapoptotic effect of ANP is primarily mediated via protein kinase A (PKA) and PI-3-kinase (PI3K)-Akt pathways [125–127]. In addition, ANP prevents dimethylnitrosamine (DMN)-induced hepatic fibrosis in rats [131] and antagonizes endothelin-1 (ET-1)-induced calcium increase and cell contraction in cultured human hepatic stellate cells (HSCs) [132]. Therefore, NPs act as an endocrine linker between the heart and liver.

Besides NPs, cardiomyocytes also secrete other peptide hormones through secretory granules, and these proteins are referred as “cardiomyokines” [8, 9]. Most of such cardiomyokines function as autocrine or paracrine factors, and several cardiomyokines target remote organs as endocrine factors, which act on not only blood vessels and kidneys, but also skeletal muscles, bone, adipose tissues, and liver [8, 14, 15, 133] (Figure 2, left). These cardiomyokines are essential cardiometabolic hormones, for example, the cardiac-specific FGF-21 overexpression mice display upregulation of plasma FGF-21 levels and show a reduction in body weight and lean body mass, whereas increasing fat mass [134]; the osteocrin secreted from the heart contributes to bone formation in zebrafish [133]. Therefore, the heart is a central regulator of metabolism and energy homeostasis in noncardiac tissues, including the liver, and this further highlights the important roles of the crosstalk between the heart and liver [117, 135].

Carlos Fernandez-Patron group has identified a unique heart secreted phospholipase A2 (sPLA2), and MMP-2 is a negative regulator of sPLA2 activity [136, 137]. Under physiological conditions, MMP-2 activity maintains low levels of certain chemokines, such as monocyte chemoattractant protein-3 (MCP-3, encoded by Ccl7, an agonist of cardiac sPLA2), by cleavage of MCP-3 at a glycine/isoleucine bond [136]. Cleaved MCP-3 binds to CC-chemokine receptors-1, -2, and -3, but no longer induces calcium fluxes or promotes chemotaxis, and instead acts as a general chemokine antagonist that dampens inflammation [136, 138]. MMP-2 deficiency (functional blockade or genetic deletion) or MCP-3 may trigger cardiac sPLA2 release from the heart, which leads to cardiac inflammation and disturb cardiac metabolic homeostasis [136, 137]. Moreover, MMP-2 deficiency causes excess sPLA2 activity, which, in turn, elevates hepatic PGE2 [137]. PGE2 is a kind of vasodilatory prostanoids, treatment with either varespladib (sPLA2 inhibitor) or indomethacin inhibited PGE2-triggered acute hypertension selectively in MMP-2^−/−^, but not in wild-type mice [137]. The cardiac sPLA2 circulates in the plasma, reaching distant target organs (e.g., the liver), contributes to the hepatic inflammatory and lipid metabolic phenotype in the liver of MMP-2 deficiency, for example, increasing liver TG and plasma very low-density lipoprotein (VLDL) TG levels [136]. Therefore, MMP-2/cardiac sPLA2 system may serve multiple purposes including signaling to the liver to modulate hepatic inflammation and lipid metabolism, and maintaining systemic blood pressure homeostasis [136].

It is known that growth hormone (GH)-IGF1 signaling is a dominant mechanism regulating postnatal mammalian growth [139–144]. GH secreted from the pituitary signals to the liver to stimulate the production of IGF1, IGF binding protein 3 (IGFBP3), and IGFBP acid-labile subunit (IGFALS) via the JAK2-STAT5 pathway [139, 145]. Circulating IGF1 forms a ternary complex with IGFBP3 and IGFALS and is a major mediator of GH's effect on mammalian postnatal body growth [139, 140, 142, 146]. Recently, Wang et al. have revealed that the levels of growth differentiation factor 15 (GDF15) in the heart and plasma are elevated in a mice model of primary pediatric cardiomyopathy with secondary failure to thrive (FTT), and the plasma GDF15 levels are elevated in children with concomitant heart disease and FTT [139]. They showed that pediatric heart disease induces GDF15 synthesis and secretion by cardiomyocytes, circulating GDF15 in turn acts on the liver to inhibit GH signaling, and specifically knockdown GDF15 in Cre^+^ cardiomyocytes by AAV9-Sico-mouse Gdf15 shRNA normalizes the circulating GDF15 levels and restores liver GH signaling, establishing GDF15 as a bona fide heart-derived hormone that negatively regulates pediatric body growth via heart-liver axis mediated to suppress hepatic GH signaling [139]. In addition, hepatic and serum GDF15 levels are increased in the nonalcoholic steatohepatitis (NASH) mouse model and in patients with NASH or advanced fibrosis [147, 148]. Using GDF15-knockout mice and liver-specific GDF15-transgenic mice, Kim et al. revealed that induction of endogenous GDF15 is a compensatory mechanism to protect against the progression of NASH [147]. However, the influence of cardiac GDF15 on NAFLD/NASH is not clear.

#### 3.2.3. The Nonsecretory Cardiac Genes Influence Liver Metabolism

There is a clear link between liver dysfunction, specifically NAFLD, and cardiac dysfunction, but new evidence suggests that the reverse is also true, such as those discussed in Section 3.2.1 [66, 135, 149]. Magida and Leinwand had demonstrated that decreased left ventricular contractile function in male, but not in female, familial hypertrophic cardiomyopathy (HCM) mice (contained two mutations, a point mutation, R403Q, and a deletion of 59 amino acids in the actin-binding site bridged by the addition of nine nonmyosin amino acids), is associated with reduced capacity for ventricular fatty acid release and uptake, thus diminishing myocardial lipid (TG and fatty acid) and ATP content [150]. However, the basis for the phenotypic gender differences in HCM mice is unclear, and whether this is related to the protective effects of estrogen (E_2_) on cardiac energetics as well as liver metabolism still needs further investigation [135]. Since the heart is a principal lipolytic organ, the proposed defects in lipid clearance by the HCM heart result in elevated the levels of oleic acid and TG in circulating VLDLs and the liver [150]. Mechanistically, the reduced expression of cardiac fatty acid translocase (CD36), lipoprotein lipase, and VLDL receptor, and the decreased activities of CD36 and its regulator AMPK in the heart are responsible for these metabolic defects in HCM mice [150]. Moreover, as the activator of CD36 expression and fatty acid uptake, the expression of myocardial transcription factor FOXO1 was also reduced in the male HCM mice [150]. HCM-induced oleic acid accumulation and PKC*α*-mediated p38 MAPK activation in the liver facilitated the phosphorylation and stabilization of hepatic peroxisome proliferator-activated receptor-*γ* coactivator-1*α* (PGC-1*α*) [150–152]. PGC-1*α* drove hepatocyte nuclear factor-4 and phosphoenolpyruvate carboxykinase (PEPCK) expression in the liver and subsequently induced PEPCK-mediated gluconeogenesis and increased blood glucose levels [150, 152]. Importantly, features of ventricular architecture and contractile dysfunction in HCM mice can be rescued either by restoring the energetic deficit at the level of the cardiomyocyte via AMPK agonist, or by blocking the deleterious elevation in hepatic glucose output using the PEPCK inhibitor 3-mercaptopicolinic acid (3-MPA) [135, 150]. Certainly, the relationship between the heart and liver is not monogamous, and other tissues, such as skeletal muscle, adipose, and pancreas, are likely to be directly affected by the elevated circulating oleic acid and VLDL TG levels [135]. Therefore, these findings raise the interesting concept that the lack of use of a specific metabolic substrate by one tissue directly affects another, perhaps revealing an intertissue homeostatic feedback mechanism [135].

The subunits of Mediator (MED) are key molecules maintaining metabolic homeostasis [40, 153]. Mediator is a multiprotein complex that acts as a bridge between DNA-bound transcription factors and RNA polymerase II (RNAPII) [153]. Mediator contains 25 (yeast) or 30 (human) subunits organized into four modules: head, middle, tail, and kinase [154, 155]. The head module together with the middle module plays an essential role during the preinitiation complex assembly, contacting the RNAPII and stabilizing its interaction with the general transcription factors, while the tail interacts with sequence-specific transcription factors [153–156]. The kinase module associates reversibly with Mediator and has negative and positive regulatory roles in transcription [154, 155]. MED1 is required for peroxisome proliferator-activated receptor (PPAR) *α*-regulated gene (including those involved in fatty acid oxidation) expression in the liver [157], and for PPAR γ‐mediated differentiation of mouse embryonic fibroblasts to adipocytes [158]; moreover, it plays important roles in regulating glucose and energy metabolism in skeletal muscle [159]. MED15 is a key effector of sterol regulatory element binding protein (SREBP)-dependent gene regulation and lipid homeostasis in metazoans [160]. Grueter et al. had revealed that heart regulates systemic energy homeostasis via MED13; MED13, in turn, is negatively regulated by a heart-specific microRNA, miR-208a [153]. They also find that MED13-dependent signaling from the heart confers leanness by enhancing metabolism in the adipose tissue and liver [40]. The interorgan communication in transgenic mice with enhanced cardiac MED13 expression (MED13cTg mice) is controlled by circulating factors that enhance white adipose tissue and liver metabolism [40]. However, the circulating factors responsible for this phenotype still need further investigation.

## 4. Perspective

In Modern Medicine, the liver and heart are anatomically and physiologically connected with each other primarily via “blood circulation.” Moreover, organokines are essential mediators of organ crosstalk between the liver and heart (the heart and liver) (Figure 2). The pathophysiological interactions between the liver and heart (the heart and liver) can be classified into three groups in Modern Medicine [17, 18, 52, 53, 106, 108]: (1) liver disease resulting from the heart disease; (2) heart disease resulting from the liver disease; (3) conditions, e.g., systemic amyloidosis, affecting the heart and the liver at the same time. In TCM, the liver and heart are physiologically connected with each other primarily via “blood movement” and “qing-zhi regulation.” Heart can modulate the pathophysiology of the liver based on the TCM theory of “illness of the child-organ involving the mother-organ,” and liver can influence the pathophysiology of the heart based on the TCM theory of “mother-organ disorder involving its child-organ,” respectively (Figure 1). Thus, the coronary heart disease can be treated from the liver based on the TCM theory of “treating the heart disease from the liver” [24–26], and NAFLD can be treated by the methods of “Xing-Qi-Huo-Xue” and “Bu-Xue” in the clinical practice of TCM [27–30]. Therefore, the evidences from TCM to Modern Medicine, and from basic research studies to clinical investigations, show that there exists interorgan crosstalk between the liver and heart (the heart and liver).

The interorgan communication between the liver and heart (the heart and liver) improves our understandings on the physiological or pathophysiological phenomena of these two organs. However, human body is a complex living organism. In addition to monogenic inherited disease, most liver and heart diseases are regulated by the complex internal environment of the body and the external environment. Therefore, both identified and unidentified cardiomyokines and hepatokines might have the interactive network during modulating liver and heart homeostasis. Besides these organokines, EVs (e.g., exosome and migrasome) are the key messengers in interorgan communication, and certain nonsecretory gene-mediated multiple signals are also important in mediating interorgan communication [5, 34–42]. However, the network and detail roles of these mediators in modulating hepatocardiac or cardiohepatic interaction still need further investigation.

TCM emphasizes steady-state balance (homeostasis) and systematization/integrity, and its treatment principle tends to individualized medicine. The basic characteristics of the theoretical system of TCM are holistic concept and treatment based on the differentiation of syndrome. How to use the philosophical thinking of TCM to promote the progress of Western Medicine is worth pondering. Moreover, it is also interesting to investigate whether the relationships between the liver and heart (the heart and liver) in TCM can be explained by Modern Medicine. Therefore, we hope that the integrated Traditional Chinese and Western Medicine will contribute to the research of the liver-heart (the heart-liver) interaction network and promote the future medical progress.

## Data Availability

The data supporting this review are from previously reported studies and datasets, which have been cited.
